# There should be a World Health Assembly resolution for malaria eradication

**DOI:** 10.1186/s12936-019-2983-8

**Published:** 2019-10-21

**Authors:** Oumar Gaye

**Affiliations:** University Cheikh Anta Diop de Dakar, Dakar, Senegal

## Abstract

Several efforts are being made now for malaria elimination with a goal for eradication. New tools and strategies are being developed and there is currently renewed political engagement and interest. Several technical groups have produced a guide on elimination for policymakers and indicated different research questions to be addressed. The World Health Assembly resolution and the United Nations General Assembly convened a high-level roundtable “From High Burden to High Impact: Getting back on track to end Malaria”. In Africa, the Head of states pronounced a vision for an Africa free of malaria and launched the slogan “*Zero malaria starts with me*”. Massive efforts to sustain research capacity in the endemic countries will be critical. It will be important to both increase domestic financing, and advocate to sustain and increase funding from major donor countries. It is unethical to continue to observe deaths of so many children in malaria endemic countries, the most vulnerable populations. Considering malaria eradication as a vision and working with all the opportunities we now have could accelerate the process. Eliminating malaria with a country regional approach and progressing step by step will give us consistent information on our way towards eradication.

## Background

During the past 5 years, there has been a dramatic reduction of malaria incidence in several countries, including several African countries; this success has led to a renewed interest in the possibility of malaria elimination with the goal of eradicating the infection. The task is not so easy due to different factors:

- malaria is complex with much heterogeneity, 6 parasite species, several vectors, variable human immunity;
- the epidemiology is dependent also on climatic, economic, socio-cultural, political factors and the performance of the health system;
- resistance of vectors and pathogens and
- insufficient financing and human resources are key elements which impact negatively the objective of elimination, and furthermore eradication.

There have historically been several control efforts, although not all were successful, for example, the Global Malaria Eradication programme during 1955–1968 and the Garki project in 1970. A new commitment was made at the Ministerial Conference on Malaria in Amsterdam in October 1992, and since then effective efforts have been made possible with the willingness of National Malaria Control Programmes. The Global Technical Strategy for Malaria 2016–2030 indicates the different steps towards elimination by ensuring universal access to malaria prevention, diagnosis and treatment and by expanding research and the strengthening the enabling environment (such as increasing funding and ensuring a robust health sector response). Several efforts are being made now and we hope that progressive and complete malaria elimination strategies, at the human level and in targeted areas, are feasible.

For the success of such a goal, new tools and strategies are being developed and there is currently renewed political engagement and interest.

Groups such as the Malaria Elimination Group (MEG), Malaria Eradication Science Alliance (MESA) and Malaria Eradication Research Agenda (MalERA) have produced a guide on elimination for policymakers and indicated different research questions to be addressed; these initiatives have led to an agenda for future malaria elimination programmes. The WHO Global Malaria Programme (GMP) and the Malaria Advisory Committee provide global recommendations aimed at policy makers and intended to drive a concerted global effort to eliminate malaria.

In the research domain different advances are being made:

*New treatments are under evaluation* OZ439/PQP, MMV 048, Tafenoquine for the radical cure of *Plasmodium vivax*, Phase 1 challenge models clinical trials, development of new radical cure single dose treatments with effect on gametocytes.

*Vaccine studies* RTS,S pilot study in Kenya, Ghana, Malawi; PfSPZ vaccine trials in Tanzania, Kenya, Mali, Burkina, Gabon, Equatorial Guinea; the adenovirus-based vaccine.

*New vector control strategies*, such as genetically modified mosquitoes through gene drives.

*New models of surveillance* Mapping and Modelling, Stratification, Genomic and Immunoepidemiologic tools such as barcodes for transmission, QT-NASBA for gametocytes submicroscopic studies, SNPs to monitor drug resistance, immunological *Plasmodium falciparum* markers, immunological vector markers based on salivary peptides.

*Other major advances* are being made in biotechnology and information technology. New delivery community engagement models now integrate Mass Drug Administration with NTDs platforms. Active Case detection MSAT (Mass Screening and Treatment) and FSAT (Focal Screening and Treatment) for hotspots with the current development of a new generation of highly sensitive elimination diagnostic tests are part of new case management initiatives. Global political, cultural and sociological aspects are being taken into account in all malaria research, as these are critical for the objective of elimination and eradication. Improvement of health systems will be fundamental to achieve eradication.

*New commitments* Several global initiatives commit to a new engagement for malaria elimination and eradication, such as the Leaders Malaria Alliance 2009, the UN General Assembly resolution in April 2011, the World Health Assembly (WHA) resolution in May 2011, and the 73rd session of the United Nations General Assembly which convened a high-level roundtable “From High Burden to High Impact: Getting back on track to end Malaria”.

African Head of states and their governments under the auspices of the African Union pronounced a vision for an Africa free of malaria in January 2017 at Addis Ababa; the Ordinary AU Assembly in 2018 at Nouakchott launched the slogan “*Zero malaria starts with me*” the new continent-wide campaign for a malaria-free Africa; the campaign empowers Africans to take a stand in the fight against the deadly disease; the Economic Community of West African States’ (ECOWAS) strategic plan and the Southern African Development Community (SADEC) strategic plan promote malaria elimination through epidemic risk and mapping, transborder interventions, the promotion of oriented research to interventions strategies. To keep malaria high on the political agenda, continued commitment must be ensured to achieve elimination by 2030 with a target of eradication by 2050.

Massive efforts to train, empower and sustain research capacity in the endemic countries will be critical. Programmes such as DELTAS Africa (Developing Excellence in Leadership, Training and Science), the European & Developing Countries Clinical Trials Partnership (EDTCP), Future Leaders – African Independent Research (FLAIR), and Africa Research Excellence Fund (AREF) are some examples dedicated to this objective.

*Funding issue* A minimum investment of US$ 6.5 billion is required annually by 2020 in order to reach the objective to 2030.

*Public engagement and advocacy* We are seeing more countries heading towards elimination. The European Region remains malaria-free. Several African countries have achieved impressive improvements in diagnostic testing and surveillance. However, many countries have reported significant increases in malaria cases, therefore it is critical to both increase domestic financing, and advocate to sustain and increase funding from major donor countries. 80% of the malaria decline between 2000 and 2015 was due to the use of long-lasting insecticidal nets (LLINs); in 2017, 203 million LLINs were delivered to Africa. Therefore, we can hope that by maintaining the momentum malaria will continue to decline substantially. We can achieve the global targets by integrating the other components such as the environment, agriculture, hygiene and sanitation, education.

Focus needs to be reoriented towards a combined strategy of treatment of infections and prevention of transmission, while new drugs and tools are developed.

Let’s promote malaria eradication; it is unethical to continue to observe deaths of so many children in malaria endemic countries, the most vulnerable populations. We may think that giving a deadline for eradication is challenging, as it could be discouraging if the target is not met; but considering it as a vision and working with all the opportunities we now have could accelerate the process. Eliminating malaria with a country-regional approach and progressing step by step will give us consistent information on our way towards eradication.

All together we can make it with WHO taking the lead.

## Data Availability

Not applicable.

